# Anomalous Origin of the Artery of Davidoff and Schechter Confirmed in a Case of Dural Arteriovenous Fistula of the Superior Sagittal Sinus

**DOI:** 10.7759/cureus.82262

**Published:** 2025-04-14

**Authors:** Masahiro Indo, Soichi Oya, Naoyuki Kakuda, Michihiro Tanaka, Shigeru Nemoto

**Affiliations:** 1 Neurosurgery, Higashi Yamato Hospital, Higashiyamato, JPN; 2 Neurosurgery, Gunma University Graduate School of Medicine, Maebashi, JPN; 3 Neurology, Higashi Yamato Hospital, Higashiyamato, JPN; 4 Neuroendovascular Surgery, Kameda Medical Center, Kamogawa, JPN; 5 Neurosurgery, Kanto Rosai Hospital, Kawasaki, JPN

**Keywords:** anomaly, artery of davidoff and schechter, dural arteriovenous fistula, dural branch, falcotentorial region

## Abstract

Objective: The artery of Davidoff and Schechter (ADS) is a dural branch originating from the second segment of the posterior cerebral artery (PCA). ADS may not be discernible even with angiography and is identified when it develops as a feeder in arteriovenous malformations (AVMs), dural arteriovenous fistulas (AVFs), or tumors. Herein, we describe a case of dural AVF of the superior sagittal sinus (SSS) that caused seizures.

Clinical presentation: A 72-year-old woman had a seizure caused by a dural AVF at the SSS with the bilateral middle meningeal and occipital arteries as major feeders and marked reflux into the bilateral cortical veins via bridging veins. The angiography revealed a dural branch from the left fourth segment (P4) of the PCA entering the falx via the medial surface of the cerebellar tentorium and feeding into the shunt, which was thought to be the anomalous origin of the ADS. Endovascular embolization resulted in a significant reduction in shunt blood flow with no complications.

Conclusion: Angiography revealed feeders from an exceedingly rare variant of ADS branching from the P4 of the PCA. Comprehending the incidence and characteristics of ADS anomalies remains inadequate because of their poor visibility in angiographic examinations of healthy individuals. Typically, the ADS supplies only the dura mater; however, it can also have branches that extend to the brain parenchyma. Thus, thorough angiographic evaluation is essential when addressing conditions in falcotentorial regions that involve ADS.

## Introduction

The artery of Davidoff and Schechter (ADS) is a dural branch of the posterior cerebral artery (PCA), first described by Wollschlaeger and Wollschlaeger in a cadaver study [1]. It typically originates from the second PCA segment (P2), runs parallel to the PCA, travels posterolaterally around the midbrain, crosses the trochlear nerve to enter the cerebellar tentorium, supplies the medial and inferior surfaces of the tentorium, and eventually turns medially, reaching the falx [2,3]. In conventional cerebral angiography, it can be difficult to delineate but can be identified as a feeder in conditions like arteriovenous malformations (AVMs), dural arteriovenous fistulas (AVFs), and tumors [2-4]. The ADS is a dural branch; however, it has been reported that it may also have branches to the midbrain and cerebellum [5]. Here, we report on a case of a superior sagittal sinus (SSS) dural AVF in which the ADS, serving as one of the feeders, originated from the fourth segment (P4) of the PCA.

## Case presentation

**Figure 1:**
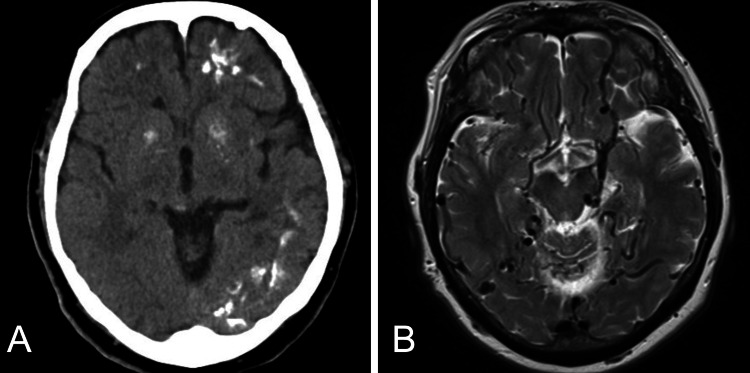
Preoperative imagings (A) Computed tomography revealing multiple calcified lesions in the left cerebral hemisphere. (B) Magnetic resonance imaging showing vein dilatation in the whole cerebrum

**Figure 2:**
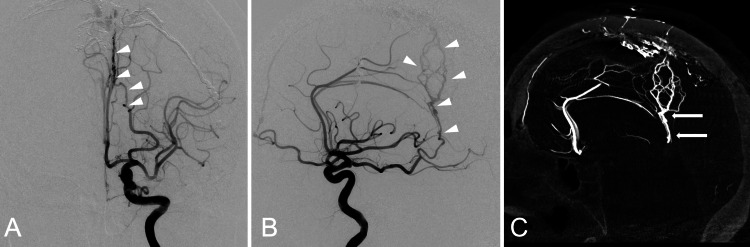
Preoperative angiograms AVF: arteriovenous fistula Left internal carotid angiograms show the anteroposterior view (A) and lateral view (B). The artery of Davidoff and Schechter (ADS) originates from the P4 of the posterior cerebral artery, running in the medial upper anterior direction. It reaches the median and then runs along the falx to the shunt point of the superior sagittal sinus (white arrowheads). The sagittal image of 3D-rotational angiography (C) shows that the ADS (white arrows) entered the cerebellar tentorium at the falcotentorial junction, running into the falx and becoming feeders of dural AVF

A 72-year-old woman without a significant medical history was transferred to our hospital because of a seizure. Computed tomography revealed numerous calcified lesions in the left cerebral hemisphere, and magnetic resonance imaging revealed marked dilatation of the cerebral veins throughout the cerebrum (Figure 1). Cerebral angiography showed a dural AVF at the SSS with the bilateral middle meningeal and occipital arteries as major feeders and marked reflux into the bilateral cortical veins via bridging veins. The diagnosis was dural AVF, Cognard type IIa+b, Borden type II. Selective left internal carotid angiography revealed a fetal posterior communicating artery and a dural branch from the left P4 entering the falx via the medial surface of the cerebellar tentorium and feeding into the shunt, which was thought to be the anomalous origin of the ADS (Figure 2). The embolization procedure was meticulously performed to prevent retrograde flow of the embolic material into the ADS, particularly due to potential small-branch occlusions invisible on pre-embolization angiography. This approach resulted in a significant reduction in the shunt blood flow. The patient’s postoperative course was favorable, and she was subsequently transferred to a rehabilitation hospital.

## Discussion

In this article, we reported the first case of a very rare vascular anomaly in which ADS originates from P4 of the PCA on cerebral angiography. Few reports have detailed the anatomical characteristics of ADS [2]. ADS on cerebral angiography was initially described by Weinstein et al. [3]. According to this report, the ADS originates from the PCA, travels around the midbrain, runs toward the falcotentorial junction, and turns anteriorly upward when it reaches the midline. However, the exact location of the origin is difficult to identify in their report. Bojanowski et al. demonstrated that the ADS originates from P2 in cerebral angiography and emphasized that the ADS is difficult to identify using conventional cerebral angiography, with confirmation only possible in diseases in which the ADS is a feeder [4]. All ADSs documented in the literature were identified as feeders for AVFs, AVMs, and tumors using cerebral angiography. There have been no reports of ADS identified on conventional cerebral angiography in patients without these abnormalities [2-4,6-8]. A medial tentorial dural branch may also originate from the superior cerebellar artery, and this dural branch is called the artery of Wollschlaeger and Wollschlaeger (AWW) [9]. In some papers, the two have been conflated and discussed [10]; however, ADS is defined as the dural branch of the PCA, which is distinct from the AWW.

ADS may be clinically significant in falcotentorial lesions. It typically does not supply brain tissue other than the dura mater, but it may provide blood supply to the vermis cerebelli and inferior colliculus [5]. Given that this anomalous ADS originated in the P4, we cautiously considered the potential risk of temporal or occipital lobe infarction during embolization. Onyx is a liquid embolic material commonly used for dural AVFs embolization. Although this allows for efficient embolization of feeders, extreme caution is necessary because it can suddenly and unpredictably flow back into numerous other branches at the shunt point. Although a previous report describes a favorable outcome from embolization via the ADS [6], caution is warranted regarding the procedure because of the risk of embolic complications from the branches supplying the brain tissue. In our opinion, ADS should be considered as a potentially hazardous anastomosis even if it is not visualized on cerebral angiography. Although this anomaly is rare, the presence of such variants can be critical during endovascular interventions to treat feeders in shunt-related conditions such as AVF and AVM of the cerebellar or falcotentorial regions.

## Conclusions

The understanding of the frequency and pattern of anomalies in ADS remains insufficient owing to their limited visibility in angiographic studies of normal patients. This report demonstrates that ADS can originate from the P4 of the PCA. In order to prevent ischemic complications during embolization in the cerebellar or falcotentorial region, it is essential to consider the potential presence of rare feeders via the falx and the cerebellar tentorium. The presence of such variants can be crucial when conducting endovascular treatments for lesions in the cerebellum or falcotentorial region.
